# Paraganglioma-induced inverted takotsubo-like cardiomyopathy leading to cardiogenic shock successfully treated with extracorporeal membrane oxygenation

**DOI:** 10.1515/med-2022-0535

**Published:** 2022-08-10

**Authors:** Fang-Fang Zhou, Jia-Sheng Ding, Min Zhang, Xin Tian

**Affiliations:** Department of Ultrasound, Lishui Central Hospital, The Fifth Affiliated Hospital of Wenzhou Medical University, Lishui 323000, Zhejiang Province, China; Department of Intensive Care Unit, Lishui Central Hospital, The Fifth Affiliated Hospital of Wenzhou Medical University, Lishui 323000, Zhejiang Province, China; Department of Pathology, Lishui Central Hospital, The Fifth Affiliated Hospital of Wenzhou Medical University, Lishui 323000, Zhejiang Province, China; Department of Intensive Care Unit, Lishui Central Hospital, The Fifth Affiliated Hospital of Wenzhou Medical University, No. 289, Kuocang Road, Lishui 323000, Zhejiang Province, China

**Keywords:** paraganglioma, extracorporeal membrane oxygenation, takotsubo cardiomyopathy, cardiogenic shock, cardiac arrest

## Abstract

Paragangliomas are rare neuroendocrine tumors that originate in the chromaffin cells of the adrenal medulla or lymph nodes. Paragangliomas manifest in rare cases as catecholamine crisis, leading to heart failure, intracranial hemorrhage, renal failure, arrhythmias, pulmonary edema, or multisystem failure. Takotsubo cardiomyopathy is also called apical ballooning syndrome or stress cardiomyopathy. Left ventricular dysfunction with apical hyperkinesis and basilar and midventricular akinesis in the absence of coronary artery disease is highly suggestive of a variant of stress cardiomyopathy (inverted takotsubo cardiomyopathy). Herein, we report the case of a 69-year-old man with an unknown retroperitoneal paraganglioma who suffered from cardiogenic shock due to inverted takotsubo cardiomyopathy. He was treated with venoarterial extracorporeal membrane pulmonary oxygenation (ECMO) in combination with an intra-aortic balloon pump. After the restoration of cardiac function, a successful transition to curative retroperitoneal paraganglioma resection was performed. We conclude that ECMO is a valuable option for undiagnosed endocrine emergencies, helping to restore cardiac function and allowing sufficient time for further accurate diagnosis and specific treatment.

## 1 Introduction

Paragangliomas are tumors that originate from paraganglial cells and can occur at any site where normal paraganglial tissue is present [1]. Approximately 10% of these tumors occur in the retroperitoneum, and the malignancy rate can be as high as 50% [2]. Functional retroperitoneal paraganglioma can secrete catecholamines and cause a series of clinical symptoms, such as paroxysmal or persistent hypertension, dizziness, headache, palpitations, excessive sweating, and occasional gastrointestinal disorders [3]. When the tumor is subjected to external effects such as compression and stress, it may suddenly release large concentrations of catecholamines, which may cause acute pulmonary edema, cardiovascular accidents, acute myocardial damage, cardiac failure, and other critical conditions [4]. Apical ballooning syndrome and stress cardiomyopathy are other names for takotsubo cardiomyopathy. The clinical presentation may be similar to that of the acute coronary syndrome; however, the disorder is defined by transient left ventricular systolic and diastolic dysfunction of the apex and midventricle in the absence of attributable coronary artery disease [5]. The inverted form has been described previously and involves basal segments with the preserved contractility of the apex [6]. In this case, a previously healthy man presented with paraganglioma-induced inverted takotsubo cardiomyopathy leading to cardiogenic shock and was treated with extracorporeal membrane pulmonary oxygenation (ECMO) as a bridge to medical treatment, followed by delayed curative retroperitoneal paraganglioma excision.

## 2 Case presentation

A 69-year-old man developed severe chest pain 23 h before without obvious cause, accompanied by sweating, chest tightness, nausea, and vomiting, and the vomiting was stomach contents. He was admitted to a local hospital, where examination showed significant ST-segment depression in leads V4–V6 and elevated troponins and was diagnosed with “acute non-ST-elevation myocardial infarction.” The patient was treated with intravenous fluid and vasodilator therapy and then transferred to our hospital for emergency admission.

He is a farmer with an active lifestyle, has received no medical care for 30 years, and has no history of long-term drug use. The patient had no history of diabetes mellitus, hyperlipidemia, hypertension, or heart disease. There was no history of abdominal surgery or trauma and no history of food or drug allergies. He smoked three to four cigarettes per day for 50 years, but he did not abuse alcohol and had never used illegal substances or dietary supplements. The patient denied having a relevant family history.

**Figure 1:**
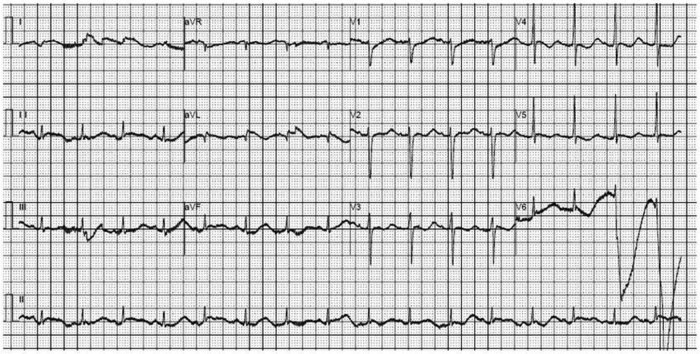
Electrocardiogram suggested mild ST-segment elevation in leads the caVL, reciprocal ST depressions in V4–V6.

**Figure 2:**
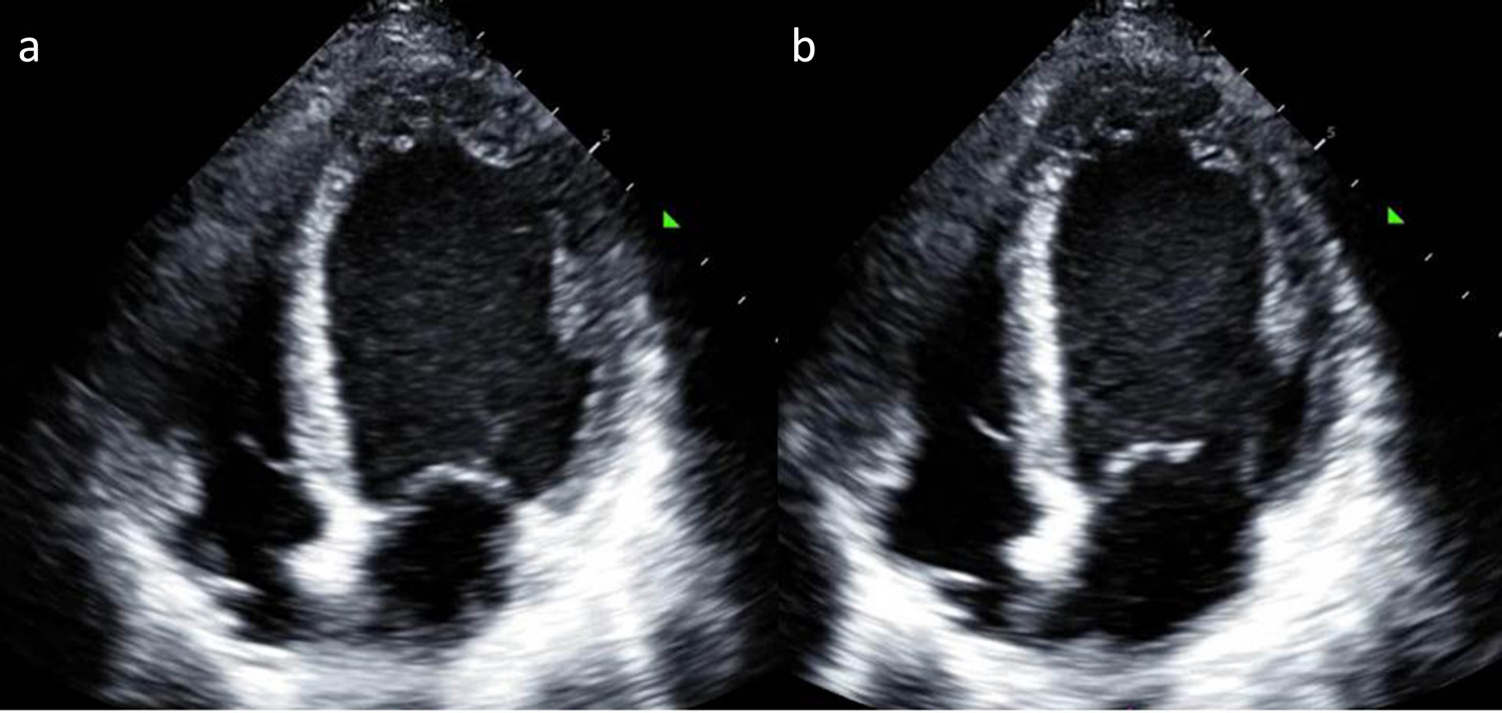
TTE. No motion in the basal and mid segments of the left ventricle but preserved apical wall motion ((a) in systole and (b) in diastole).

On admission, he was unconscious, pale, irritable, and had cold hands and feet. Physical examination showed dilated jugular veins, a grade 3 systolic murmur could be heard in the apical region, and inspiratory wet rales were observed in both lungs. The abdomen was soft, without pressure, rebound pain, or muscle tension, and there was no palpable swelling of the liver or spleen. There was no edema in either lower extremity, and no abnormal neurological signs were observed. Blood pressure was 105/83 mmHg on norepinephrine maintenance. Heart rate was 145 beats/min on adrenaline maintenance. The electrocardiogram suggested mild ST-segment elevation in leads I and aVL, reciprocal ST depressions in V4–V6 (Figure 1). Arterial blood gas analysis revealed hypoxemia (PaO_2_, 50 mmHg) and metabolic acidosis (pH, 7.276; PaCO_2_, 33.5 mmHg; bicarbonate, 15 mmol/L; lactate, 7.7 mmol/L). The troponin T concentration was 49.90 ng/mL, and myoglobin was >2000.0 ng/mL. Further laboratory studies showed renal insufficiency (creatinine, 240 μmol/L; urea nitrogen, 11.7 μmol/L) and hyperglycemia (glucose, 15.26 mmol/L). Further cardiac evaluation was needed, including transthoracic echocardiography (TTE) and coronary angiography, to rule out obstructive coronary disease. The patient was then wheeled into the catheterization laboratory for coronary angiography, during which he went into cardiac arrest. Cardiopulmonary resuscitation was initiated, and he underwent tracheal intubation and received epinephrine, sodium bicarbonate, and atropine. Autonomic circulation was restored, and postintubation blood pressure was 130/88 mg Hg with a heart rate of 126 beats per minute. Coronary angiography suggested no coronary artery stenosis. TTE revealed a left ventricular ejection fraction (LVEF) of 10% with no motion in the basal and mid-segments of the left ventricle but preserved apical wall motion (Figure 2). The right ventricular size was normal. The left atrium was mildly enlarged. There was no pericardial effusion. The absence of acute plaque rupture or obstructive coronary artery disease and the pattern of left ventricular dysfunction (preserved apical contractility and basilar hypokinesis) arouse suspicion for takotsubo cardiomyopathy. Although apical ballooning is the most common pattern of stress cardiomyopathy, apical hyperkinesis and basilar hypokinesis have been described in other cases (so-called inverted takotsubo cardiomyopathy). Takotsubo cardiomyopathy commonly manifests as dyspnea, syncope, or chest pain, although patients may also have a sudden cardiac arrest, heart failure, or cardiogenic shock, as seen in this patient. Over the next hour, progressive shock developed in the patient despite vasopressor support with norepinephrine, epinephrine, and dopamine. Because the patient’s circulatory failure was expected to be recoverable, the hospital’s ECMO team was on standby before intubation. In the catheterization laboratory, we inserted an intra-aortic balloon pump (IABP) catheter and then supported the patient with ECMO. Twenty minutes later, he was transferred to the intensive care unit.

After resuscitation, the patient’s temperature was 38.4°C, blood pressure was maintained at 100/60 mmHg under epinephrine and norepinephrine micropump injections, and respiratory rate was 30 breaths per minute. His pupils were bilaterally dilated, and pupillary light reflexes were absent. The oxygen saturation was 95% while he was receiving 80% oxygen from a volume-control ventilator with a tidal volume of 500 ml and a positive end-expiratory pressure of 5 cmH_2_O. Therapeutic hypothermia was implemented for neuroprotection. This therapy is used to minimize neurologic injury in patients after an arrest who are unable to follow commands or make purposeful movements or in whom the neurologic status cannot be reliably assessed, as was the case for this patient. A repeated TTE 10 hours after the initial evaluation showed a significant decrease in left ventricular systolic function (visually estimated LVEF, 10–15%), no motion in the basal or mid-left ventricular segments, preserved apical wall motion, and mild mitral regurgitation (grade ¼). The transaortic flow had fully ceased. The patient’s 24-h urine metanephrine and epinephrine levels measured 95 μg (reference range: 10–80 μg/24 h) and 35 μg (reference range: <20 μg/24 h), respectively. Even though the patient was being treated with norepinephrine, these findings were regarded as symptoms of pheochromocytoma or paraganglioma.

TTE on day 5 with an ECMO flow rate of 2.0 L/min indicated normal left ventricular activity (LVEF, 65%) without any regional wall motion anomalies, indicating that the patient’s cardiac status had significantly improved. With the support of ECMO and IABP, the hemodynamic situation improved, and vasoactive drugs were rapidly reduced. Cardiac function improved rapidly based on repeated TEE studies. The LVEF recovered from an initial 10–15 to 65% in just 5 days. ECMO support was withdrawn on day 7 after admission, IABP assistance was withdrawn on day 9, and tracheal intubation was removed on day 11.

**Figure 3:**
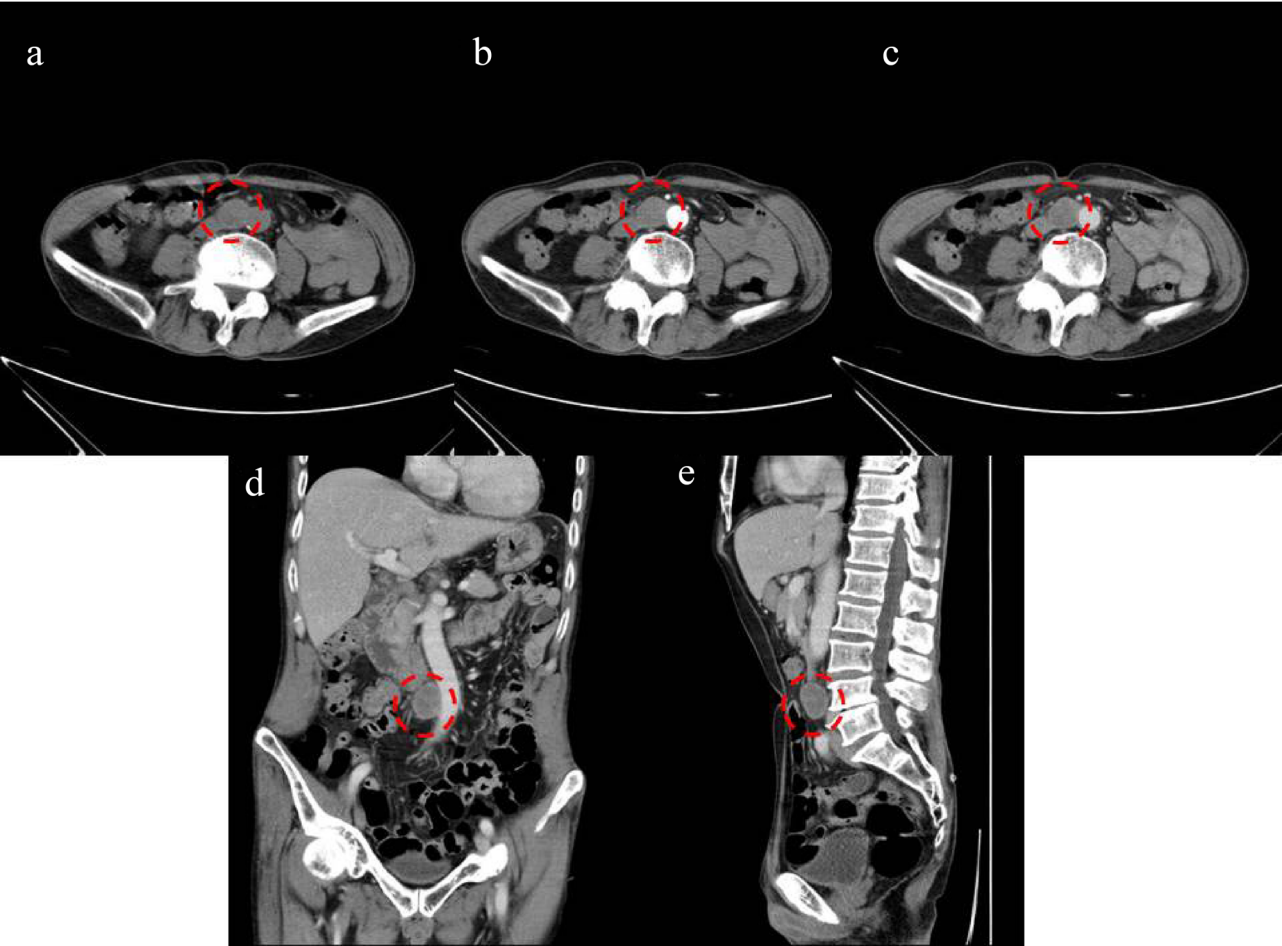
Contrast-enhanced CT of the abdomen. (a) Noncontrast-enhanced CT scan revealed an isointense solid mass with a clear boundary adjacent to the abdominal aorta (red dashed circle); (b and c) arterial and venous phase enhancement scans with mild enhancement of the mass (red dashed circle); (d and e) multiplanar reconstructed sagittal and coronal views revealed the adjacent relationship between the mass and the surrounding structures (red dashed circle).

**Figure 4:**
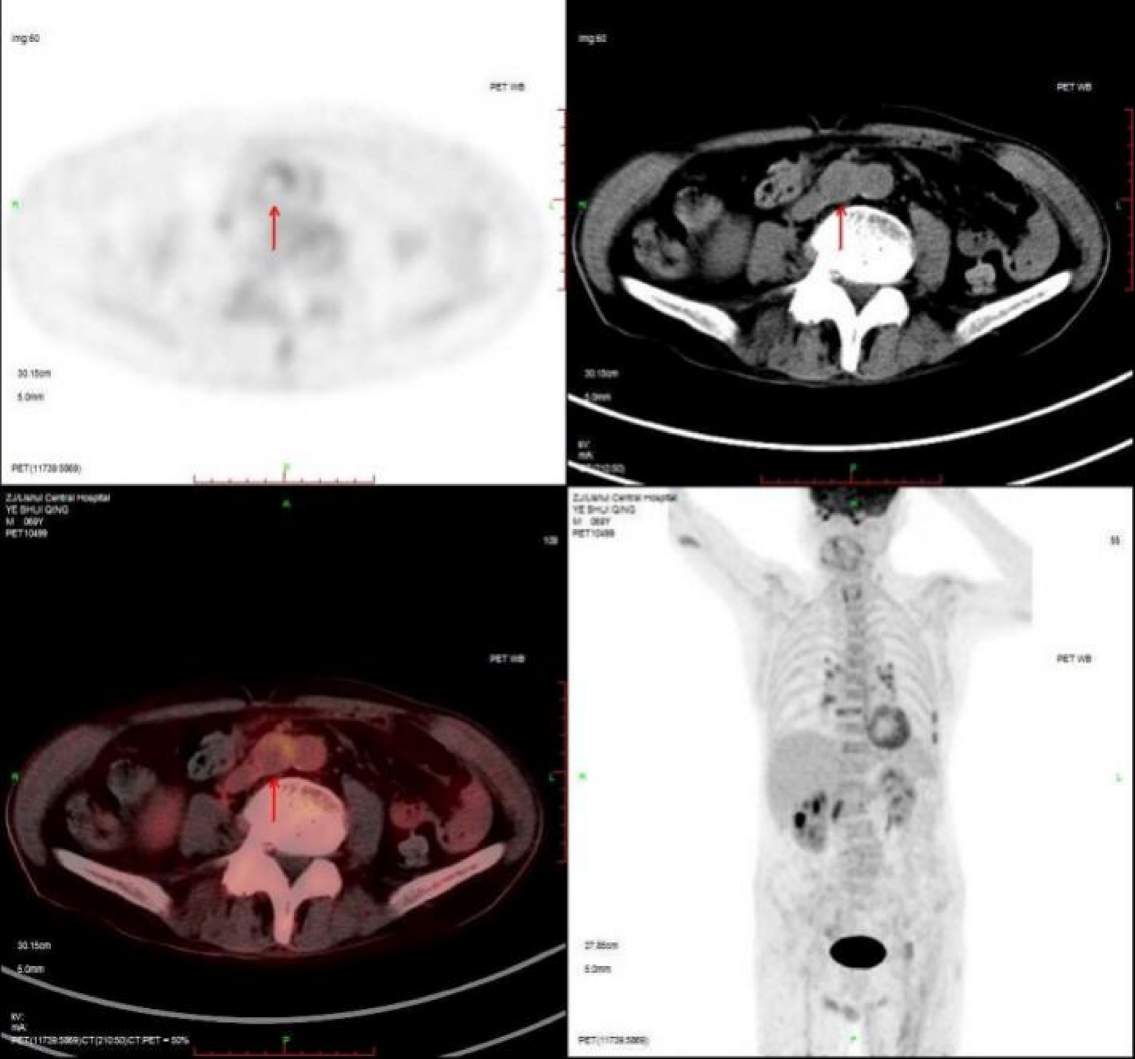
PET-CT scan revealed an isointense mass with a clear boundary near the abdominal aorta with mild uptake of the mass (red arrow).

A subsequent abdominal contrast-enhanced computed tomographic (CT) scan showed a 30 mm × 29 mm × 24 mm solid mass adjacent to the abdominal aorta that was isointense with clear boundaries and mild enhancement on an enhanced scan (Figure 3). A positron emission tomography computed tomography (PET-CT) scan suggested a retroperitoneal paraganglioma (Figure 4).

**Figure 5:**
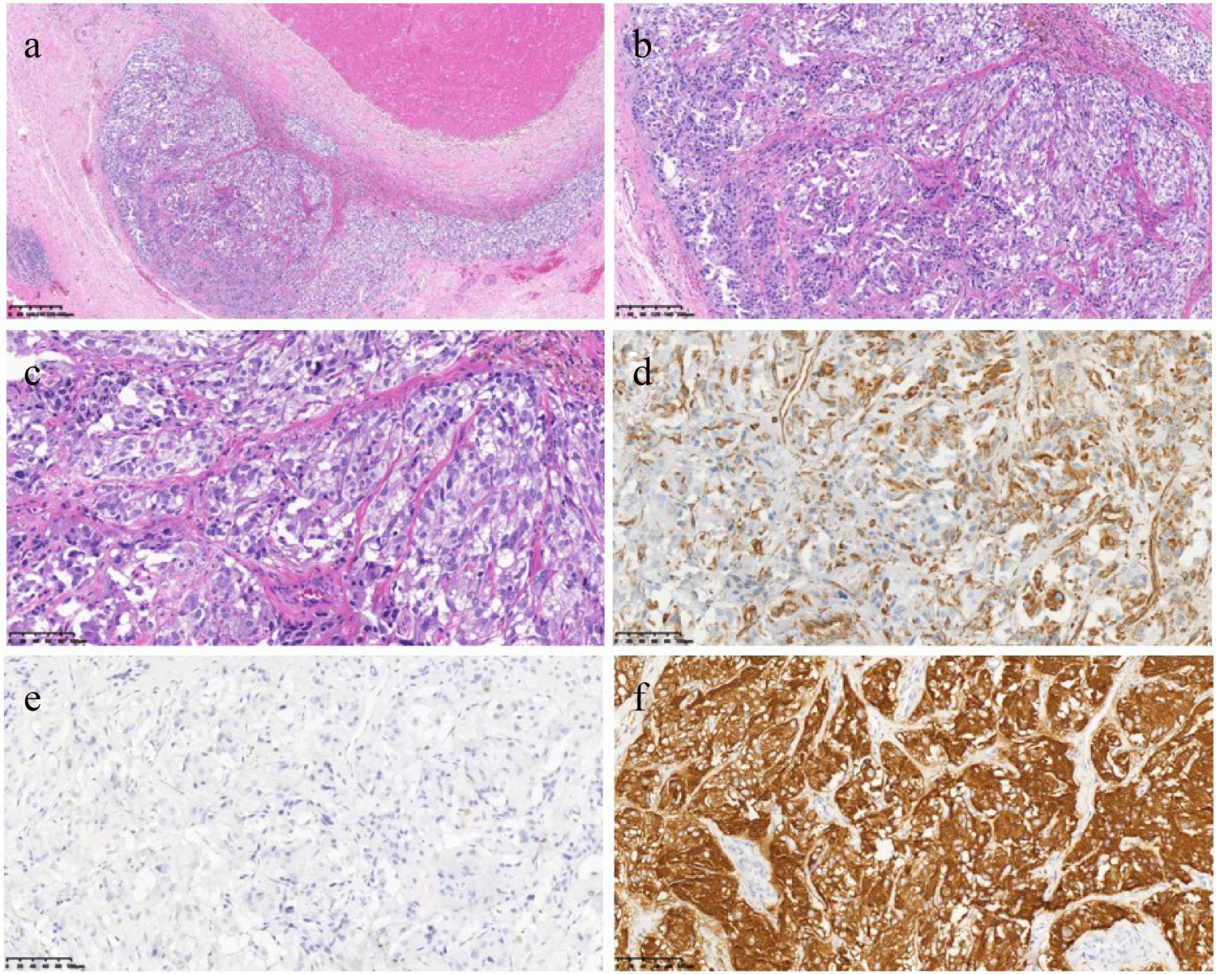
Histopathological findings and immunohistochemical staining. (a) Hematoxylin–eosin (HE) staining (×40); (b) He staining (×100); (c) HE staining (×200); (d) immunostaining for vimentin (×100); (e) immunostaining for CK (×100); (f) immunostaining for Syn (×100).

Three months after weaning from ECMO, the patient underwent retroperitoneal tumor resection. Postoperative pathology suggested that small clusters of mildly morphologic epithelioid tumor cells were seen at the margins of a large area of necrotic tissue, with no significant vascular or nerve involvement. Immunohistochemical staining showed that the tumor cells were positive for Vim, S100, Ki67 (2% positive), Syn and CgA, but negative for CK (Figure 5). They confirmed the diagnosis of paraganglioma.

The patients are started on trimetazidine after discharge to promote myocardial metabolism and myocardial energy production while reducing cardiac workload. He was then followed up every 6 mo, and each follow-up examination included a physical examination; assessment of plasma catecholamine, urinary catecholamine, and VMA levels; an abdomen CT; and a TTE. The findings suggested that he did not require further cardiac medications. There was no local or systemic recurrence after 2 years of surgical resection.


**Statement of Ethics:** Ethics approval was obtained from the Ethics Committee of Lishui Central Hospital. Informed consent was waived because of the retrospective nature of the study and our Ethics Committee also approved the informed consent waiver. The study protocol was in accordance with the Declaration of Helsinki.
**Consent for publication:** Written informed consent for publication was obtained from the patient.

## 3 Discussion

Retroperitoneal paragangliomas originate from retroperitoneal sympathetic paraganglial tissue and account for 1–3% of retroperitoneal tumors [2]. They occur in patients aged 30–50 years, have no significant sex differences, and are mostly functional tumors [7]. They can occur anywhere in the retroperitoneum, most commonly in the organ of Zuckerkandl, between the origin of the inferior mesenteric artery and the bifurcation of the abdominal aorta [8]. When functional retroperitoneal paraganglioma is subjected to external stimuli such as compression and stress, it may suddenly release large concentrations of catecholamines, which may cause several acute cardiovascular syndromes, including stress (takotsubo) cardiomyopathy and myocardial infarction [4].

The international consensus on takotsubo cardiomyopathy was published in 2014, which stated that takotsubo-like cardiomyopathy should be mentioned in the presence of explanations such as pheochromocytoma [9]. Inverted takotsubo cardiomyopathy was first reported in 2005. This rare variant has been mentioned several times since then, and these cases describe reversible hypokinesis in the mid and basal segments with preserved apical contractility [10]. In the absence of coronary artery disease, left ventricular failure with apical hyperkinesis and basilar and midventricular akinesis, as seen in our patient, is strongly suggestive of inverted takotsubo cardiomyopathy.

The definitive treatment for paraganglioma is surgical resection [11]; however, in this case, our patient presented with acute refractory shock, and the priority was to restore cardiac function and reverse myocardial damage as soon as possible. For refractory cardiac shock, mechanical life support with ECMO is an effective treatment, especially when heart failure is potentially reversible. This patient presented with refractory cardiac shock characterized by extremely low cardiac output with a LVEF of only 10–15%. In such life-threatening circumstances, IABP and V-A ECMO can be utilized as extracorporeal support systems to stabilize their hemodynamics and allow sufficient time for subsequent treatment. IABP provides only a limited amount of additional cardiac output, but this is usually not sufficient for acute refractory shock. V-A ECMO provides adequate perfusion to all organs regardless of the lung condition. It can perform both ventricular and pulmonary functions and can support a failing heart for as long as needed. In this case, MAP increased to 65 mm Hg immediately after initiation of V-A ECMO. IABP was also used to reduce cardiac afterload, ensure coronary perfusion, and maintain pulse pressure during the early stages of V-A ECMO. After initiation of V-A ECMO and IABP, patient organ perfusion improved, metabolic acidosis was corrected, and the vasoactive drug response was restored. The patient’s vital signs were stable with ECMO and IABP support. Subsequently, a retroperitoneal paraganglioma was found on a contrast-enhanced abdominal CT scan. It is usually recommended to perform imaging to identify pheochromocytoma or paraganglioma only after the biochemical results suggest the diagnosis. However, for critically ill patients with high suspicion, we recommend early imaging examinations because the results of biochemical tests are delayed and the high likelihood that the elevated levels are due to physiologic stress and vasopressor use.

Tumor resection was performed 3 months after ECMO. Postoperative histological examination showed that the tumor was a paraganglioma with extensive necrosis. We suspect that the release of large concentrations of catecholamines was due to hemorrhagic necrosis of the tumor, which subsequently caused cardiogenic shock in the patient. The pathology confirmed our suspicions.

To date, including this patient, we found only 23 [12,13,14,15,16,17,18,19,20,21,22] reports of ECMO for the treatment of cardiogenic shock due to paraganglioma or pheochromocytoma. Among them, there were 21 cases of pheochromocytoma and 2 cases of paraganglioma. The clinical characteristics of these cases are summarized in Table 1. Of the 19 patients treated with ECMO in Table 1, 16 patients (including one maternal) recovered stable vital signs with ECMO support and underwent subsequent tumor resection. Three patients died due to the development of irreversible multiorgan failure. Catastrophic shock is completely reversible and does not lead to further damage to the myocardium if ECMO therapy is provided in a timely manner and for a sufficiently long period of time during the acute phase. When patients have unstable hemodynamics, mechanical life support with ECMO should be given as soon as possible to prevent the development of irreversible multiorgan failure.

**Table 1:** Summary of the reported clinical characteristics of ECMO for the treatment of cardiogenic shock due to paraganglioma- or pheochromocytoma-induced cardiogenic shock

Characteristic		*n* (%)
Age (year)		23
	≤30	4 (17.4)
	30–60	17 (73.9)
	≥60	2 (8.7)
Sex		23
	Male	9 (39.1)
	Female	14 (60.9)
Location		23
	Adrenal	21 (91.3)
	Extra-adrenal	2 (8.7)
Tumor size (mm)		17
	≤40	7 (41.2)
	40–80	7 (41.2)
	≥80	3 (17.6)
Medical history		20
	Palpitations	6 (30.0)
	Headache	7 (35.5)
	Chest pain	9 (45.0)
	Hypertension	5 (25.0)
Pre-ECMO LVEF (%)		18
	≤10	2 (11.1)
	10–30	14 (77.8)
	≥30	2 (11.1)
ECMO duration (days)		23
	≤5	13 (56.6)
	5–10	7 (30.4)
	≥10	3 (13.0)
Surgical approach		19
	Yes	16 (84.2)
	No	3 (15.8)
Status		19
	ANED	16 (84.2)
	DOD	3 (15.8)

ANED, alive with no evidence of disease; DOD, dead of disease; ECMO, extracorporeal membrane oxygenation; LVEF, left ventricular ejection fraction.

## 4 Conclusion

This patient suffered cardiogenic shock due to inverted takotsubo cardiomyopathy, which was eventually found to be caused by excessive catecholamine secretion from retroperitoneal paraganglioma. We conclude that catecholamine-induced inverted takotsubo cardiomyopathy appears to be reversible and can be cured with prompt and adequate treatment. ECMO mechanical life support is a valuable option for the treatment of inverted takotsubo cardiomyopathy, helping the patient recover cardiac function. Paraganglioma should systematically be considered for patients with unexplained cardiogenic shock.

## References

[1] Jeevan DS, Saleh M, LaBagnara M, Neil JA, Hillard VH. Malignant carotid body tumor presenting with myelopathy: case report. J Neurosurg Spine. 2016;24(4):660–3. 10.3171/2015.8.SPINE13483 26722959

[2] Chrisoulidou A, Kaltsas G, Ilias I, Grossman AB. The diagnosis and management of malignant phaeochromocytoma and paraganglioma. Endocr Relat Cancer. 2007;14(3):569–85. 10.1677/ERC-07-0074 17914089

[3] Lenders JW, Eisenhofer G, Mannelli M, Pacak K. Phaeochromocytoma. Lancet. 2005;366(9486):665–75. 10.1016/S0140-6736(05)67139-5 16112304

[4] Favier J, Amar L, Gimenez-Roqueplo AP. Paraganglioma and phaeochromocytoma: from genetics to personalized medicine. Nat Rev Endocrinol. 2015;11(2):101–11. 10.1038/nrendo.2014.188 25385035

[5] Templin C, Ghadri JR, Diekmann J, Napp LC, Bataiosu DR, Jaguszewski M, et al. Clinical features and outcomes of takotsubo (Stress) cardiomyopathy. N Engl J Med. 2015;373(10):929–38. 10.1056/NEJMoa1406761 26332547

[6] Song BG, Chun WJ, Park YH, Kang GH, Oh J, Lee SC, et al. The clinical characteristics, laboratory parameters, electrocardiographic, and echocardiographic findings of reverse or inverted takotsubo cardiomyopathy: comparison with mid or apical variant. Clin Cardiol. 2011;34(11):693–9. 10.1002/clc.20953 PMC6652294 22031226

[7] Neumann HPH, Young WF, Jr., Eng C. Pheochromocytoma and paraganglioma. N Engl J Med. 2019;381(6):552–65. 10.1056/NEJMra1806651 31390501

[8] Kahraman D, Goretzki PE, Szangolies M, Schade H, Schmidt M, Kobe C. Extra-adrenal pheochromocytoma in the organ of Zuckerkandl: diagnosis and treatment strategies. Exp Clin Endocrinol Diabetes. 2011;119(7):436–9. 10.1055/s-0030-1270511 21374546

[9] Redfors B, Shao Y, Lyon AR, Omerovic E. Diagnostic criteria for takotsubo syndrome: a call for consensus. Int J Cardiol. 2014;176(1):274–6. 10.1016/j.ijcard.2014.06.094 25043217

[10] Ennezat PV, Pesenti-Rossi D, Aubert JM, Rachenne V, Bauchart JJ, Auffray JL, et al. Transient left ventricular basal dysfunction without coronary stenosis in acute cerebral disorders: a novel heart syndrome (inverted takotsubo). Echocardiography. 2005;22(7):599–602. 10.1111/j.1540-8175.2005.40046.x 16060897

[11] Asa SL, Ezzat S, Mete O. The diagnosis and clinical significance of paragangliomas in unusual locations. J Clin Med. 2018;7(9):280. 10.3390/jcm7090280 PMC6162705 30217041

[12] Choudhary M, Chen Y, Friedman O, Cuk N, Ben-Shlomo A. Pheochromocytoma crisis presenting with ARDS successfully treated with ECMO-assisted adrenalectomy. AACE Clin Case Rep. 2021;7(5):310–4. 10.1016/j.aace.2021.03.008 PMC8426613 34522771

[13] Sojod G, Diana M, Wall J, D’Agostino J, Mutter D, Marescaux J. Successful extracorporeal membrane oxygenation treatment for pheochromocytoma-induced acute cardiac failure. Am J Emerg Med. 2012;30(6):1017-e1. 10.1016/j.ajem.2011.05.006 21741786

[14] Suh IW, Lee CW, Kim YH, Hong MK, Lee JW, Kim JJ, et al. Catastrophic catecholamine-induced cardiomyopathy mimicking acute myocardial infarction, rescued by extracorporeal membrane oxygenation (ECMO) in pheochromocytoma. J Korean Med Sci. 2008;23(2):350–4. 10.3346/jkms.2008.23.2.350 PMC2526426 18437026

[15] Chao A, Wang CH, You HC, Chou NK, Yu HY, Chi NH, et al. Highlighting indication of extracorporeal membrane oxygenation in endocrine emergencies. Sci Rep. 2015;5:13361. 10.1038/srep13361 PMC4547135 26299943

[16] Flam B, Broome M, Frenckner B, Branstrom R, Bell M. Pheochromocytoma-induced inverted takotsubo-like cardiomyopathy leading to cardiogenic shock successfully treated with extracorporeal membrane oxygenation. J Intensive Care Med. 2015;30(6):365–72. 10.1177/0885066614552992 25286918

[17] Kiamanesh O, Vu EN, Webber DL, Lau E, Kapeluto JE, Stuart H, et al. Pheochromocytoma-induced takotsubo syndrome treated with extracorporeal membrane oxygenation: Beware of the apical sparing pattern. JACC Case Rep. 2019;1(2):85–90. 10.1016/j.jaccas.2019.06.001 PMC8301256 34316755

[18] Zhou X, Liu D, Su L, Long Y, Du W, Miao Q, et al. Pheochromocytoma crisis with severe cyclic blood pressure fluctuations in a cardiac pheochromocytoma patient successfully resuscitated by extracorporeal membrane oxygenation: a case report. Medicine (Baltimore). 2015;94(17):e790. 10.1097/MD.0000000000000790 PMC4603056 25929929

[19] Bouabdallaoui N, Bouchard D, Jolicoeur EM, Chronopoulos A, Garneau PY, Lamarche Y. Extracorporeal membrane oxygenation in pheochromocytoma-induced cardiogenic shock. Asian Cardiovasc Thorac Ann. 2018;26(4):314–6. 10.1177/0218492317727995 28823181

[20] van Zwet CJ, Rist A, Haeussler A, Graves K, Zollinger A, Blumenthal S. Extracorporeal membrane oxygenation for treatment of acute inverted takotsubo-like cardiomyopathy from hemorrhagic pheochromocytoma in late pregnancy. A A Case Rep. 2016;7(9):196–9. 10.1213/XAA.0000000000000383 27607406

[21] Hekimian G, Kharcha F, Brechot N, Schmidt M, Ghander C, Lebreton G, et al. Extracorporeal membrane oxygenation for pheochromocytoma-induced cardiogenic shock. Ann Intensive Care. 2016;6(1):117. 10.1186/s13613-016-0219-4 PMC5126035 27896787

[22] Montalto A, Nicolo F, Polizzi V, Comisso M, Musumeci F. Fast myocardial recovery ensured by the combined use of V-A ECMO and IMPELLA CP in cardiogenic shock related to a pheochromocytoma crisis. J Card Surg. 2020;35(9):2367–9. 10.1111/jocs.14805 32720331

